# Gender specific cut-off points of age for disability among rural elderly in Anhui Province, China

**DOI:** 10.3389/fpubh.2022.945849

**Published:** 2022-10-04

**Authors:** Xinran He, Xianwen Wang, Min Zhang, Weizheng Zhu, Yuyang Liu, Qian Sun, Guimei Chen, Min Li, Hong Ding

**Affiliations:** ^1^School of Health Management, Anhui Medical University, Hefei, China; ^2^School of Public Health and Health Management, Anhui Medical College, Hefei, China

**Keywords:** disability, age, ROC curve, elderly, Chinese

## Abstract

**Objective:**

The purpose of this study was to determine the optimal cut-off values of age for disability in order to predict the risk of disability for older adults in rural areas.

**Methods:**

WHO Disability Assessment Schedule 2.0 was used to assess disability. The cut-off values of age for disability were obtained by ROC curve analysis.

**Results:**

The cut-off points of age for cognition restriction, mobility restriction, self-care restriction, getting along with people restriction, life activities restriction, and social participation restriction for men were 70.5, 68.5, 72.5, 70.5, 71.5, and 68.5 years old, respectively. The cut-off points of age for cognition disability, mobility restriction, self-care disability, getting along with people disability, life activities disability, and social participation disability for women were 72.5, 71.5, 70.5, 70.5, 71.5, and 71.5 years old, respectively. Over the cut-off values of age was an independent risk factor for disability (*P* < 0.05).

**Conclusion:**

Presenting first disability symptoms were different between men and women. Preventive efforts to prevent future disability should be different for men and women.

## Introduction

A significant proportion of older adults living in rural areas experience functional limitations resulting in disability. Several investigations on older people living in rural China indicated that daily activities disability rate was up to 28.4% (1), cognition disability rate was up to 16.7% (2), and social participation disability rate was up to 20.84% (3). Older adults experiencing disability may place significant strain on the health care system, long-term care system, and health insurance (4, 5). Average demand for medical expenses of one disabled elder is 1000 yuan higher than that of the non-disabled elderly in China (6). In addition to its financial impact, individuals with disability face higher rates of poverty, loss of independence, and health challenges (4, 7).

The high burden and cost of disability makes it imperative to understand the age of disability onset and take interventions before then. Existing research on disability of older adults mainly discussed the influencing factors, illustrating that aging, being female, low economic income, low educational level, living alone, absence of health insurance, residence in a rural area, depression, frailty, and higher number of hospitalization were risk factors (8–11). However, these studies just pointed out that the risk of disability increases with aging. The question of what specific age disability will onset has not been addressed. Also, gender differences in disability suggested that age for disability onset might be also different between male and female (12). Although substantial studies have been done regarding risk factors of disability, there is a critical lack of research concerning gender specific age for disability onset in the Chinese rural elderly population.

ROC curve analysis originated from the theory of electronic signal detection in the early 1950s (13), and has since been applied to psychology and clinical diagnoses (14). ROC curve can be used to evaluate the effectiveness of predictors in identifying the diseased and non-diseased population, and the optimal cut-off value can be determined by the maximum Youden index. The individuals on one side of the optimal cut-off value were marked as diseased, and the individuals on the other side were marked as healthy (15). It is suggested that we could identify the optimum cut-off values of age for disability onset with the help of ROC curve analysis.

WHO redefines disability as a general term including physical structure and functional impairment, daily activities restriction, and social participation restriction in the *International Classification of Functioning, Disability, and Health (ICFDH)* (16). A variety of tools can be used to evaluate disability status for older people, such as WHO Disability Assessment Schedule 2.0 (WHO DAS 2.0) (17), Activities of Daily Living (ADL) (18), and Short Form-36 (SF-36) (19). The theoretical framework of ADL and SF-36 were not consistent with the structure and content of ICFDH, which cannot comprehensively and accurately evaluate disability. According to the classification structure and content of ICFDH, WHO DAS 2.0 developed in 2001 can comprehensively evaluate the overall function of older adults in six dimensions.

Effective prevention and care of disability requires suitable measures tailored for elderly people. An understanding of gender specific cut-off values of age for disability onset would facilitate the implementation of more appropriate interventions. Therefore, we used WHO DAS 2.0 to measure disability. The gender specific cut-off values of age for disability onset were determined by ROC curve analysis and possible reasons for differences were explored in discussion. Suggestions of prevention and care for disability based on gender specific cut-off values of age for disability onset were provided.

## Materials and methods

### Data and sample

From January to July in 2018, a multi-stage stratified cluster random sampling survey method was adopted. According to the geographical location, economic development, and other factors, we divided Anhui Province into three regions: Northern Anhui Province, Central Anhui Province, and Southern Anhui Province. A county was randomly selected from each region as a sample county. Two towns were selected randomly from each county, three villages were selected randomly from each town, a total of 18 villages were selected as survey points. A total of 125 households were randomly selected from each village. In the households survey, all older adults aged 60 years old and above were participants. Inclusive criteria: (1) age ≥ 60 years old, (2) voluntary participation in the survey, and (3) registered residence in Anhui Province and living in the local area for more than 1 year. For older adults with cognitive impairment, language communication impairment, and hearing impairment, their family members or caregivers who were familiar with them could answer the questionnaire on their behalf. Exclusion criteria: people with cognitive impairment, hearing impairment, language communication impairment, and no family members or caregivers who were familiar with them who could answer the questionnaire on their behalf.

The survey was conducted with the assistance of local healthcare committees, village committees, and village doctors. The questionnaire surveys were completed by face-to-face interviews with specially trained investigators at the participants' home. A total of 3,491 older people were interviewed and 3,336 valid questionnaires were obtained. The effective response rate was thus 95.56% (3,336/3,491).

### Assessment instruments

#### WHO disability assessment schedule 2.0 (WHO DAS 2.0)

WHO DAS 2.0 is a generic scale for measuring disability, which consists of 36 items, divided into 6 domains: (1) cognition, (2) mobility, (3) self-care, (4) getting along with people, (5) life activities, (6) social participation (20). Each item includes five levels: 1=no difficulty, 2=mild, 3=moderate, 4=severe, 5=extreme/unable to do. If participants choose 2, 3, 4, 5, the item will be identified as a disability. As long as one item of the participant is identified as a disability, the participant is judged as disabled (21). This test has high reliability and good validity (22).

#### Demographic characteristics

The participants' key demographic information was collected, including gender, age, education level, economic status, profession status, living arrangement, number of chronic diseases, and hospitalization within the previous year.

#### Statistical analysis

Epidata3.1 (EpiData Association, Odense, Denmark) was used to establish the database. Data were entered twice by two trained people independently and checked for consistency. SPSS 17.0 (SPSS, Inc., Chicago, IL, USA) was used to analyze the data. Descriptive analysis was used to describe the social demographic characteristics of the participants. Chi square test and rank sum test were used to compare the differences in social demographic characteristics by genders. Area under curve (AUC), sensitivity, specificity, and Youden index (Youden index = sensitivity + specificity-1) were calculated by SPSS 17.0. If the AUC > 0.5, the ROC curve has predictive power. The corresponding age of the maximum Youden index was considered the best cut-off values. Logistic regression model was used to verify the effect of cut-off values of age on disability. We used a significance level of alpha = 0.05.

## Results

### Sample characteristics

In total, 3,336 respondents comprised the effective sample, including 1,640 males and 1,696 females.

Table 1 describes the sample characteristics by genders. The average age of the participants was 71.17 ± 7.103, with no significant difference between men (71.25 ± 6.775) and women (71.10 ± 7.408). The proportion of illiteracy of females (84.5%) was significantly higher than that of males (47.4%). The proportion of poverty in the male group (39.3%) was significantly higher than that in the female group (33.1%). A total of 64.1% of the participants were unemployed, and the proportion of unemployed participants in the female group (70.5%) was significantly higher than that in the male group (57.4%).

**Table 1:** Sample characteristic by genders, *N* (%).

	**Total (*N =* 3,336)**	**Male (*N =* 1,640)**	**Female (*N =* 1,696)**	***P-*value**
Educational level				< 0.001
Illiterate	2,211 (66.3)	778 (47.4)	1,433 (84.5)	
Elementary school or higher	1,125 (33.7)	862 (52.6)	263 (15.5)	
Economic status				< 0.001
Impoverished	1,206 (36.2)	645 (39.3)	561 (33.1)	
Non-impoverished	2,130 (63.8)	995 (60.7)	1,135 (66.9)	
Profession				< 0.001
Unemployed	2,137 (64.1)	941 (57.4)	1,196 (70.5)	
Employed	1,199 (35.9)	699 (42.6)	500 (29.5)	
Living arrangement				0.004
Alone	660 (19.8)	306 (18.7)	354 (20.9)	
With a spouse	1,436 (43.0)	753 (45.9)	683 (40.3)	
Others	1,240 (37.2)	581 (35.4)	659 (38.9)	
Chronic diseases				< 0.001
0	865 (25.9)	473 (28.8)	392 (23.1)	
1	1,227 (36.8)	631 (38.5)	596 (35.1)	
≥2	1,244 (37.3)	536 (32.7)	708 (41.7)	
Hospitalization				0.548
Yes	1,062 (31.8)	514 (31.3)	548 (32.3)	
No	2,274 (68.2)	1,126 (68.7)	1,148 (67.7)	

Additionally, 19.8% of the participants were living alone and 43.0% were living with a spouse. Moreover, a total of 74.1% of the participants had one and more chronic diseases. Finally, 31.8% of the participants had been hospitalized during the year before the survey, and there were no significant differences between the male and female groups.

### Cut-off points of age for disability

**Figure 1:**
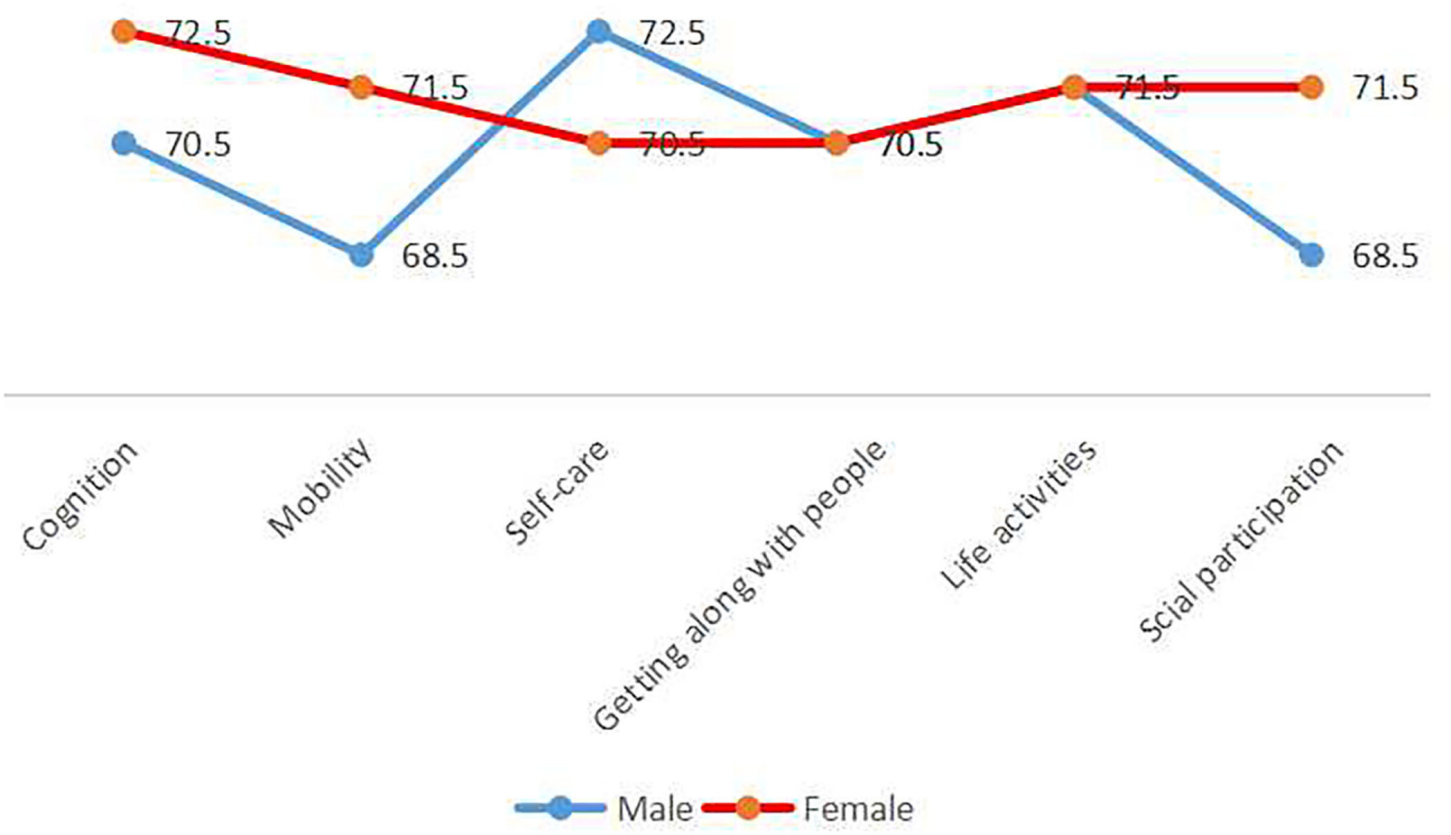
Gender specific cut-off points of age for disability.

The optimum cut-off points of age for disability were identified by ROC curves analysis. The cut-off points are showed in Table 2 and  Figure 1. For cognition, the cut-off values of age were found to be 70.5 years old among men and 72.5 years old among women, the sensitivity and specificity were 53.2 and 59.3% for men and 42.5 and 76.5% for women (*p* < 0.05). With regard to mobility, at cut-off levels of 68.5 years old for men and 71.5 years old for women, the sensitivity and specificity were 68.8 and 52.3% among men and 48.8 and 77.1% among women (*p* < 0.05). Considering self-care, the cut-off values were as follows 72.5 years old for men and 70.5 years old for women, the sensitivity and specificity were 58.6 and 32.9% for men and 64.0 and 62.5% for women (*p* < 0.05). The optimum cut-off values of age for getting along with people were both 70.5 years old for men and women, and the sensitivity and specificity were 62.8% and 51.8 among men, and 60.3 and 63.5% among women (*p* < 0.05). The optimum cut-off values of age for life activities were 71.5 years old for both men and women, and the sensitivity and specificity were 53.9 and 68.9% among men, and 49.0 and 75.4% among women (*p* < 0.05). For social participation, the cut-off values of age for men was 68.5 years old with 62.3% sensitivity and 55.5% specificity, and for women was 71.5 years old with 43.0% sensitivity and 74.3% specificity (*p* < 0.05).

**Table 2:** Gender specific cut-off points of age for disability.

	**Male**					**Female**				
	**Cut-off**	**Sensitivity**	**Specificity**	**AUC (95%*CI*)**	***P***	**Cut-off**	**Sensitivity**	**Specificity**	**AUC (95%*CI*)**	***P***
**Cognition**	70.5	53.2	59.3	57.8 (54.9~60.6)	< 0.001	72.5	42.5	76.5	62.2 (59.3~65.1)	< 0.001
**Mobility**	68.5	68.8	52.3	64.0 (61.4~66.7)	< 0.001	71.5	48.8	77.1	67.1 (64.3~69.9)	< 0.001
**Self-care**	72.5	58.6	32.9	66.6 (63.5~69.7)	< 0.001	70.5	64.0	62.5	68.6 (65.9~71.3)	< 0.001
**Getting along with people**	70.5	62.8	51.8	63.4 (60.5~66.3)	< 0.001	70.5	60.3	63.5	66.4 (63.8~69.0)	< 0.001
**Life activities**	71.5	53.9	68.9	64.6 (62.0~67.3)	< 0.001	71.5	49.0	75.4	67.0 (64.3~69.7)	< 0.001
**Social participation**	68.5	62.3	55.5	61.3 (57.3~65.3)	< 0.001	71.5	43.0	74.3	61.0 (55.7~66.3)	< 0.001

Cut-off values of age are expressed in years. Sensitivities, specificities, and AUCs are expressed in percentage (%).

### Effects of cut-off values of age on disability

The effects of cut-off values of age for disability were showed in Table 3. Logistic regression analysis was carried out with the six dimensions disability status (no disability = 0, disability = 1) as dependent variables, and the cut-off values of age (≤ cut-off value of age =1, >cut-off value of age =2) as independent variables. Over cut-off value of age was independent risk factor for disability, the minimum OR is 1.817, the maximum OR is 2.890, and the differences were statistically significant (*P* < 0.05).

**Table 3:** Effects of cut-off values of age for disability in terms of six dimensions by genders.

	**Male**					**Female**				
	***B***	***SE***	***Wald***	***OR (95%CI)***	***P-Value***	***B***	***SE***	***Wald***	***OR (95%CI)***	***P-Value***
**Cognition**	0.617	0.115	28.775	1.853 (1.479~2.322)	< 0.001	1.061	0.136	60.649	2.890 (2.212~3.774)	< 0.001
**Mobility**	0.729	0.113	41.973	2.074 (1.663~2.586)	< 0.001	1.055	0.136	59.991	2.871 (2.199~3.750)	< 0.001
**Self-care**	0.786	0.127	38.486	2.196 (1.713~2.815)	< 0.001	0.951	0.115	68.461	2.589 (2.067~3.243)	< 0.001
**Getting along with people**	0.632	0.117	29.355	1.881 (1.497~2.365)	< 0.001	0.823	0.109	56.965	2.276 (1.839~2.819)	< 0.001
**Life activities**	0.849	0.116	53.448	2.337 (1.862~2.935)	< 0.001	1.046	0.130	65.130	2.846 (2.207~3.668)	< 0.001
**Social participation**	0.626	0.165	14.395	1.870 (1.353~2.584)	< 0.001	0.597	0.241	6.141	1.817 (1.133~2.913)	0.013

Statistic results were obtained after adjusting educational level, economic status, profession, living arrangement, chronic diseases, and hospitalization.

## Discussion

This study was conducted to develop gender specific cut-off points of age for disability for rural older adults in Anhui Province, so as to determine their risk of disability.

This study has demonstrated that age is an important variable to influence and predict the risk of disability, and it interacts with gender. As one ages, health problems accumulate and function declines and risk of non-communicable diseases increases (23), which increases their disability risk. There were some differences in presenting first disability symptoms and cut-off points of age for disability in terms of cognitive, mobility, self-care, and social participation between male and female. This indicates the health services that men and women of the same age require might be different.

The first disability symptoms presenting in men were mobility and social participation related, which indicated that the dysfunctions of mobility and social participation were the precursor of male disability, and they were targets for intervention to delay disability with effect. A prior study demonstrated a significant association between mobility limitation and participation restriction (24). On the one hand, older adults with mobility limitations might feel more unsafe in their neighborhood, and were less likely to walk around, potentially affecting social participation (25). On the other hand, social isolation may decrease musculoskeletal function, accelerating the mobility limitation relevant to aging (26). One disability does not worsen independently, but also causes another disability.

The first disability symptoms presenting in women were in self-care and getting along with others, which indicates that dysfunctions of self-care and getting along with others were the precursor of female disability, providing intervention in those areas first may improve women's performance effectively (27). Most rural women spent their entire life engaging in daily activities and housework. Self-care required the joint participation of multiple organs of the body, which may be the most complex activity for rural women, so their self-care ability declined first.

Whether male or female, the earliest presenting disability symptom was not single. Our study represented that two dimensions of disability emerged at the same time. Which indicated that a single or independent intervention may not be effective in preventing or delaying disability onset. The intervention program should be designed based on gender and two presenting symptoms, forming a multiple and comprehensive program to improve older adults' functions better.

The cut-off value of age for self-care disability of women was earlier than that of men. This finding was consistent with the result of a previous study (28). The effect of gender difference on onset of age for self-care disability might be affected by educational level. The higher cut-off point of age of males was due to higher educational level of males who participated in the study. Older adults with higher educational levels might have better judgment and decision-making to perform self-care behaviors (29).

Except for the cut-off value of age for self-care disability, the cut-off values of age for disability of cognition, mobility, getting along with people, life activities, and social participation for women was either equal to or higher than men. The earliest cut-off value of age for disability for men was 2 years earlier than that for women, which meant that men would enter the high-risk of disability period earlier than women. Men were more likely to drink, smoke, and perform hazardous driving behavior than women, which led to a high incidence rate and prevalence of cardiovascular and other chronic non-communicable diseases and men were more prone to violence and road injuries (30–32).

Our results showed that gaps between the onset of one disability and the next was small, the longest gap was 2 years, and the shortest gap was just merely 1 year, this has profound implications. Care delivery institutions offering higher quality of care need to take into account that older adults' functional capabilities may be getting worse during their period of care, and care programs have to be changed accordingly to adapt to such dynamic realities.

In conclusion, this study took older people in rural regions as research subjects, and explored the cut-off values of age for disability onset. The data may be used by researchers as reference data and by policy makers as reference indexes for formulating policies of healthcare for Anhui Province or central China. The limitations of mobility and social participation were the two first presenting symptoms for men. The restrictions on self-care and getting along with people were the two first presenting symptoms for women. The nursing homes can incorporate interventions to prevent future disability according to first presenting disability symptoms by genders.

There were some limitations in this study. First, we only studied the cut-off values of age for disability of older people in rural areas of Anhui Province. In the future, samples from other provinces in China and other countries should be included in the study. Second, this study was a cross-sectional survey, and the age of disability onset was not obtained.

## Conclusions

Presenting first disability symptoms were different between men and women. Age at onset of disability for men was earliest for mobility and social participation while age for women was earliest for self-care and getting along with people. The preventive efforts need to be targeted at earlier ages in order to prevent future disability and they should be different for men and women.

## Data availability statement

The datasets generated during the study are not publicly available due to an ethical restriction but are available from the corresponding author on reasonable request.

## Ethics statement

The studies involving human participants were reviewed and approved by the Research Ethics Committee of Anhui Medical University. Written informed consent for participation was not required for this study in accordance with the national legislation and the institutional requirements. All participants were fully informed about the study purpose and methods and provided verbal informed consent.

## Author contributions

XH conceptualized the study and wrote the article. XW, MZ, WZ, YL, and QS contributed to the study design, data collection and data processing, and statistical analysis. XW contributed to the literature review. XH, XW, GC, ML, and HD revised the article. All authors contributed to the article and approved the submitted version.

## Funding

This study was funded by Research Projects of Humanities and Social Sciences in Colleges and Universities of Anhui Province (No. SK2018A0165) and Doctoral Fund Project of Anhui Medical University (No. XJ201545). The funders had no role in the design of the study and collection, analysis, and interpretation of data and in writing the manuscript.

## Conflict of interest

The authors declare that the research was conducted in the absence of any commercial or financial relationships that could be construed as a potential conflict of interest.

## Publisher's note

All claims expressed in this article are solely those of the authors and do not necessarily represent those of their affiliated organizations, or those of the publisher, the editors and the reviewers. Any product that may be evaluated in this article, or claim that may be made by its manufacturer, is not guaranteed or endorsed by the publisher.

## References

[1] Xu R Mao K Wang K Gong F Zhang J Wang W. Status of activities of daily living among the elderly in rural areas. Chin J Gerontol. (2019) 39:5837–40. 10.3969/j.issn.1005-9202.2019.23.057

[2] Yang X Yang X Yang J Qu F Huang Y Chen H. Effects of arteriosclerosis on mild cognitive impairment in the elderly in rural areas of Guizhou Province. Chin J Gerontol. (2022) 42:3594–7. 10.3969/j.issn.1005-9202.2022.14.061

[3] Shi X Zhu C Zhen P Tian Q. Epidemiology and influencing factors of social participation ability of rural elderly in Henan Province. J Zhengzhou Univ. (2021) 56:568–72. 10.13705/j.issn.1671-6825.2021.01.099

[4] Jehn A Zajacova A. Disability trends in Canada: 2001-2014 population estimates and correlates. Can J Public Health. (2019) 110:354–63. 10.17269/s41997-018-0158-y 30547289 PMC6964543

[5] Lin YH Chen YC Tseng YC Tsai ST Tseng YH. Physical activity and successful aging among middle-aged and older adults: a systematic review and meta-analysis of cohort studies. Aging. (2020) 12:7704–16. 10.18632/aging.103057 32350152 PMC7244057

[6] Bai L Gu S Gu H Xu X Nan C Li D. The impact of disability on intergenerational care needs of the elderly in China. Inquiry J Health Car. (2021) 58:469580211018283. 10.1177/00469580211018283 34027690 PMC8150436

[7] Wang X Sun M Li X Lu J Chen G. Effects of disability type on the association between age and non-communicable disease risk factors among elderly persons with disabilities in Shanghai, China. Int J Environ Res Public Health. (2020) 17:5426. 10.3390/ijerph17155426 32731459 PMC7432529

[8] Chen S Qin J Li Y Wei Y Long B Cai J. Disability and its influencing factors among the elderly in a county, Guangxi Province, China. Int J Environ Res Public Health. (2018) 15:1967. 10.3390/ijerph15091967 30205622 PMC6163965

[9] Ran L Kong H Du M He J Zhong Q Ran Y. Comparison of health-related quality of life between the Han and Yi ethnicity elderly in the Yi autonomous areas of Yunnan Province. BMC Geriatr. (2019) 19:326. 10.1186/s12877-019-1257-1 31766992 PMC6878633

[10] Liu H Jiao J Zhu C Zhu M Wen X Jin J. Potential associated factors of functional disability in Chinese older inpatients: a multicenter cross-sectional study. BMC Geriatr. (2020) 20:319. 10.1186/s12877-020-01738-x 32883253 PMC7650523

[11] Qiao R Jia S Zhao W Xia X Su Q Hou L. Prevalence and correlates of disability among urban-rural older adults in Southwest China: a large, population-based study. BMC Geriatr. (2022) 22:517. 10.1186/s12877-022-03193-2 35739469 PMC9229854

[12] Carmona-Torres JM Rodríguez-Borrego MA Laredo-Aguilera JA López-Soto PJ Santacruz-Salas E Cobo-Cuenca AI. et al. Disability for basic and instrumental activities of daily living in older individuals. PLoS ONE. (2019) 14:e0220157. 10.1371/journal.pone.0220157 31348797 PMC6660130

[13] Swets JA. Indices of discrimination or diagnostic accuracy: their ROCs and implied models. Psychol Bull. (1986) 99:100–17. 10.1037/0033-2909.99.1.100 3704032

[14] Hajian-Tilaki K. Receiver Operating Characteristic (ROC) Curve analysis for medical diagnostic test evaluation. Caspian J Intern Med. (2013) 4:627–35. 24009950 PMC3755824

[15] Ruopp MD Perkins NJ Whitcomb BW Schisterman EF. Youden Index and optimal cut-point estimated from observations affected by a lower limit of detection. Biom J. (2008) 50:419–30. 10.1002/bimj.200710415 18435502 PMC2515362

[16] World Health Organization. International Classification of Functioning, Disability and Health (ICF). (2001). Available online at: https://www.who.int/classifications/icf/en/ [accessed April 10, 2022.]

[17] Üstün TB Chatterji S Kostanjsek N Rehm J Kennedy C Epping-Jordan J. Developing the World Health Organization disability assessment schedule 2.0. Bull World Health Organ. (2010) 88:815–823. 10.2471/BLT.09.067231 21076562 PMC2971503

[18] Xu R Zhou X Cao S Huang B Wu C Zhou X. Health status of the elderly and its influence on their activities of daily living in Shangrao, Jiangxi Province. Int J Environ Res Public Health. (2019) 16:1771. 10.3390/ijerph16101771 31109138 PMC6572997

[19] Brazier J Roberts J Deverill M. The estimation of a preference-based measure of health from the SF-36. J Health Econ. (2002) 21:271–92. 10.1016/S0167-6296(01)00130-8 11939242

[20] Linares-Moya M Rodríguez-Torres J Heredia-Ciuró A Granados-Santiago M López-López L Quero-Valenzuela F. Psychological distress prior to surgery is related to symptom burden and health status in lung cancer survivors. Support Care Cancer. (2022) 30:1579–1586. 10.1007/s00520-021-06537-7 34541609 PMC8727403

[21] Xenouli G Xenoulis K Sarafis P Niakas D Alexopoulos EC. Validation of the World Health Organization Disability Assessment Schedule (WHO-DAS II) in Greek and its added value to the Short Form 36 (SF-36) in a sample of people with or without disabilities. Disabil Health J. (2016) 9:518–23. 10.1016/j.dhjo.2016.01.009 26996759

[22] Haylett R Gustafson O. A feasibility study to assess pre-admission status and six month outcomes of major trauma patients admitted to an intensive care unit, using the WHO DAS 20. J Crit Care. (2018) 48:140–4. 10.1016/j.jcrc.2018.08.030 30193172

[23] Fong JH. Disability incidence and functional decline among older adults with major chronic diseases. BMC Geriatr. (2019) 19:323. 10.1186/s12877-019-1348-z 31752701 PMC6873710

[24] Liu JY. The severity and associated factors of participation restriction among community-dwelling frail older people: an application of the International Classification of Functioning, Disability and Health (WHO-ICF). BMC Geriatr. (2017) 17:43. 10.1186/s12877-017-0422-7 28143597 PMC5286833

[25] Hand CL Howrey BT. Associations among neighborhood characteristics, mobility limitation, and social participation in late life. J Gerontol B Psychol Sci Soc Sci. (2019) 74:546–55. 10.1093/geronb/gbw215 28158866 PMC6377035

[26] Liu X Yu HJ Gao Y Zhou J Zhou M Wan L. Combined association of multiple chronic diseases and social isolation with the functional disability after stroke in elderly patients: a multicenter cross-sectional study in China. BMC Geriatr. (2021) 21:495. 10.1186/s12877-021-02439-9 34530729 PMC8447675

[27] Henskens M Nauta IM Drost KT Scherder EJ. The effects of movement stimulation on activities of daily living performance and quality of life in nursing home residents with dementia: a randomized controlled trial. Clin Interv Aging. (2018) 13:805–17. 10.2147/CIA.S160031 29750023 PMC5933359

[28] Ning P Du E Du C Meng Q Wu B. Prevalence of the disability and inflection point age of the disability level change, Shandong Province. Prev Med. (2020) 47:1633–64.

[29] Tabrizi JS Behghadami MA Saadati M Söderhamn U. Self-care ability of older people living in urban areas of Northwestern Iran. Iran J Public Health. (2018) 47:1899–905. 30788305 PMC6379604

[30] Karriker-Jaffe KJ Tam CC Cook WK Greenfield TK Roberts S. Gender equality, drinking cultures and second-hand harms from alcohol in the 50 US States. Int J Environ Res Public Health. (2019) 16:4619. 10.3390/ijerph16234619 31766337 PMC6926546

[31] Kyu HH Abate D Abate KH Abay SM Abbafati C Abbasi N. Global, regional, and national disability-adjusted life-years (DALYs) for 359 diseases and injuries and healthy life expectancy (HALE) for 195 countries and territories, 1990-2017: a systematic analysis for the Global Burden of Disease Study 2017. Lancet. (2018) 392:1859–922. 10.1016/S0140-6736(18)32335-3 30415748 PMC6252083

[32] Cislaghi B Weber AM Gupta GR Darmstadt GL. Gender equality and global health: intersecting political challenges. J Glob Health. (2020) 10:010701. 10.7189/jogh.10.010701 32257161 PMC7101083

